# Rash and Heart Block: A Unique Case of Lyme Carditis

**DOI:** 10.7759/cureus.21332

**Published:** 2022-01-17

**Authors:** Yasmeen M Daraz, Omar Abdelghffar

**Affiliations:** 1 Internal Medicine, Montefiore Medical Center, New York City, USA; 2 Internal Medicine, Elmhurst Hospital Center, New York City, USA

**Keywords:** erythema migrans, tick-borne illness, wenckebach, atrioventricular heart block, carditis, lyme's disease

## Abstract

Lyme disease is a multisystem disease that can present as a life-threatening condition known as Lyme carditis. While most commonly manifesting as a fluctuating atrioventricular block, Lyme carditis can also emerge as myocarditis and coronary artery events. This case report will detail the clinical scenario of a 23-year-old patient who presented with acute onset fluctuating atrioventricular block and erythema migrans and was found to have Lyme carditis. The patient was treated promptly with antibiotics, thus avoiding long-term Lyme disease sequela, with a complete resolution of his disease, including his high degree atrioventricular block.

## Introduction

The most common tick-borne illness in the United States is Lyme disease [1]. In North America, infection is mostly caused by *Borrelia burgdorferi*, where Lyme carditis occurs in approximately 1% of patients with Lyme disease [2]. Lyme disease manifests as a multisystem disease that is divided into (1) days to weeks after infection presenting with erythema migrans and general flu-like symptoms, (2) weeks to months after infection with neurologic or cardiac symptoms, and (3) months to years after infection with arthritis and neurological symptoms [2]. Patients with cardiac involvement may present with symptoms of lightheadedness, syncope, shortness of breath, palpitations, and/or chest pain. Therefore it is important to obtain an EKG and monitor for varying degrees of atrioventricular block, which is the most common cardiac manifestation of Lyme carditis. Prompt monitoring and treatment of Lyme carditis are essential to prevent potentially severe cardiac arrhythmias and later complications of Lyme disease.

## Case presentation

**Figure 1:**
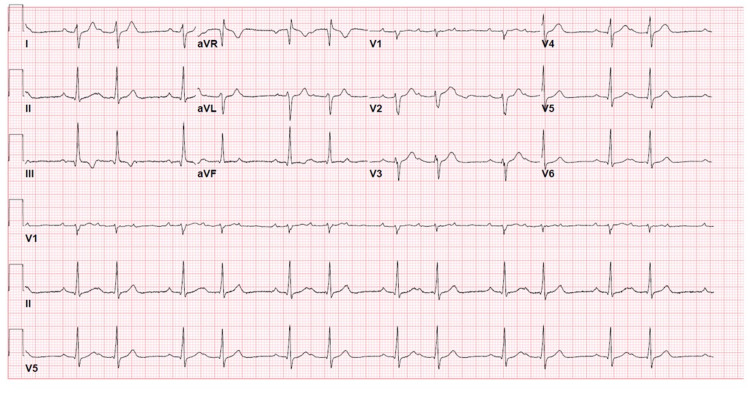
Mobitz Type 1 Second Degree Block

A 23-year-old male with a past medical history of obesity presented to the emergency room with a chief complaint of palpitations for three days. Other associated symptoms included weakness, episodic postural dizziness, and chest tightness. His symptoms continued to worsen as the days progressed with worsening chest tightness on inspiration. Other pertinent history included a hiking trip in Nyack, New York, three to four weeks prior to presentation. Initial vitals were a temperature of 100.3 F, heart rate 94 bpm, respiratory rate of 18, blood pressure of 136/83, and oxygen saturation of 98% on room air. On physical exam, he was noted to have numerous targetoid multi-centimeter rashes with central clearing most consistent with erythema migrans on his trunks and extremities. Cardiac enzymes were normal, as well as thyroid stimulating hormone. Chest x-ray and transthoracic echocardiogram showed no abnormal findings. Initial EKG showed a Mobitz Type 1 second degree block (Figure 1).

**Figure 2:**
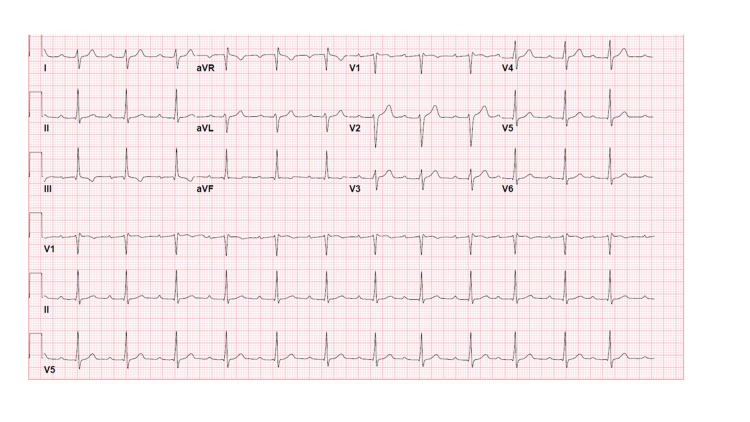
Progression to First Degree Atrioventricular Block

Parenteral ceftriaxone 2 grams daily and acetaminophen 650 mg every six hours as needed were started empirically until Lyme serologies resulted. Blood work showed positive IgG and IgM antibody titers, as well as a negative coronavirus disease 2019 (COVID-19) test. The patient was admitted to a telemetry unit where he improved in rhythm from Mobitz Type 1 second degree block to first degree atrioventricular block after four days of parenteral ceftriaxone, with concomitant improvement in his erythema migrans rash and fever (Figure 2). He was discharged on a 14-day course of doxycycline 100 mg twice a day.

**Figure 3:**
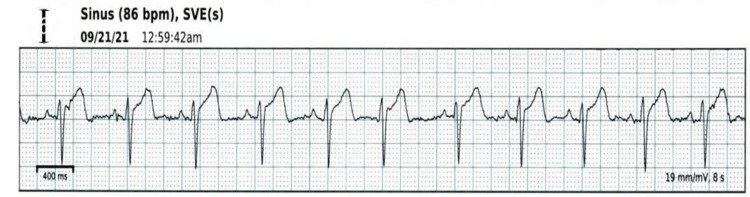
Zio® Patch Showing Normal Sinus Rhythm

Three weeks after discharge, the patient was seen in an electrophysiology clinic where EKG was noted to be normal sinus rhythm. Zio® Patch (iRhythm Technologies, Inc., San Francisco, California, United States) was applied to the patient to confirm resolution, which showed the predominant underlying rhythm to be normal sinus rhythm (Figure 3).

## Discussion

Lyme disease is a systemic disease caused by the spirochete *B. burgdorferi* and transmitted by the Ixodes tick [3]. The three stages are characterized by an influenza-like illness, usually accompanied by an erythema migrans rash, to neurologic and cardiac sequelae. Cardiac manifestations predominantly affect the conduction system and myocardium, which can present with conduction derangements most commonly involving the atrioventricular node. Other cardiac manifestations of Lyme carditis include myocarditis, pericardial effusion, acute coronary events, coronary artery aneurysm, tachyarrhythmias, and congestive heart failure [4]

Diagnosing Lyme carditis requires a clinical association between a patient’s history and his or her clinical and laboratory data. A negative enzyme-linked immunoassay (ELISA) serologic test for the presence of *B. burgdorferi* antibodies does not rule out the possibility of Lyme carditis due to the low negative predictive value of this test. Studies show that the ELISA serologic test can be negative in the first six to eight weeks of disease [5].

While Lyme carditis has been shown to be self-limited, antibiotic therapy in the early stages of Lyme disease prevents its later complications [6]. Oral antibiotics with amoxicillin or doxycycline are sufficient in patients with minor cardiac involvement. However, patients with second- or third-degree atrioventricular conduction block with a PR interval greater than 300 ms, require hospitalization in a telemetry unit for intravenous administration of ceftriaxone or high dose penicillin G [7]. Oral antibiotic can be started once the PR interval decreases to <300 ms, but parenteral antibiotic should be continued until the high-degree atrioventricular block has resolved [7].

This case demonstrates the importance of promptly treating Lyme carditis when suspicion is high, based on a culmination of clinical and historical data. While a temporary pacemaker may be required for advanced heart block, our patient’s high-degree atrioventricular block resolved with prompt antibiotic treatment and admission to the telemetry unit for close monitoring [8]. Patients who present with a high degree atrioventricular block should always have Lyme serologies checked and included in the differential diagnosis. A low threshold to start prophylactic antibiotic treatment prevents unnecessary pacemaker insertion and later complications of Lyme disease.

## Conclusions

This case report illustrates the importance of promptly diagnosing and treating Lyme carditis with antibiotics to prevent long-term complications. Lyme carditis may initially only present with nonspecific symptoms such as shortness of breath, fatigue, or palpitations, as seen in this case. Therefore, there should be a low threshold to obtain an electrocardiogram as well as Lyme serologies - especially if presenting in a Lyme endemic area. Once a high-degree atrioventricular block is found, history and clinical data points should be thoroughly assessed to prevent life-threatening conditions of Lyme carditis or the need for a permanent pacemaker.
